# Nucleation Enhancement in Stereodefective Poly(l-lactide) by Free Volume Expansion Resulting from Low-Temperature Pressure CO_2_ Preconditioning

**DOI:** 10.3390/polym10020120

**Published:** 2018-01-26

**Authors:** Qiaofeng Lan, Jian Yu, Jun Zhang, Jiasong He

**Affiliations:** 1 School of Biomedical Engineering, Southern Medical University, Guangzhou 510515, China; 2 Beijing National Laboratory for Molecular Sciences, CAS Key Laboratory of Engineering Plastics, Institute of Chemistry, Chinese Academy of Sciences (CAS), Beijing 100190, China; jzhang@iccas.ac.cn (J.Z.); hejs@iccas.ac.cn (J.H.)

**Keywords:** poly(l-lactide), carbon dioxide, nucleation, crystallization, free volume

## Abstract

Nucleation enhancement in a highly stereodefective poly(l-lactide) (PLLA) with an optical purity of 88% by low-temperature pressure (0 and 35 °C under 2 MPa) CO_2_ preconditioning was investigated using differential scanning calorimetry (DSC), infrared (IR) spectroscopy, polarized optical microscopy (POM) as well as positron annihilation lifetime spectroscopy (PALS). Despite the preconditioning of the melt-quenched films for 2 h, IR results indicated that no trace of mesophase was generated and the samples remained in the glassy state. However, judging from the results of DSC, IR, and POM, when compared to the untreated sample, both the treated ones showed a significantly enhanced crystal nucleation effect, resulting in the corresponding greatly enhanced crystallization kinetics. Moreover, owing to the existence of the retrograde vitrification, the conditions of the previous low-pressure CO_2_ conditioning affected the nucleation enhancement effect. When compared to the case of 35 °C, the much lower temperature of 0 °C was more effective for nucleation enhancement. The PALS results indicated that the enlarged free volume, which resulted from the CO_2_ conditioning, largely accounted for the formation of locally ordered structures, providing many more potential nucleation sites for forming critical nuclei and thus the resulting enhanced crystallization kinetics in glassy PLLA. The present results have implications in understanding the nucleation enhancement effect, in particular in stereodefective PLLA systems, which possess extremely low crystallization ability and are thus probably too problematic to be evaluated by conventional methods.

## 1. Introduction

Poly(l-lactide) (PLLA) has attracted much attention, not only because it can be derived from renewable resources but also because it has attractive properties such as biodegradability, high biocompatibility, and good processability [1,2]. Physical properties of PLLA largely depend upon crystalline structures [3]. Unfortunately, compared to its petrochemical-derived counterparts, the restricted crystallization ability of PLLA limits its large-scale application. Particularly, the crystallization rate of PLLA decreases dramatically with the decrease of optical purity [4]. Several strategies have been extensively utilized to accelerate the crystallization of PLLA, such as incorporation of heterogeneous nucleating agents, addition of plasticizing agents, and a combination of both approaches [5,6,7]. The poly(d-lactide) (PDLA) has also been beneficially applied for the formation of the stereocomplex of PLLA/PDLA as a special nucleating agent, resulting in a speeded-up crystallization rate as well as the enhanced melting temperature of polylactide crystals [8,9]. Additionally, long-time (ca. 10^1^–10^3^ h) physical aging at temperatures around the glass transition temperature (*T*_g_) contributes to the enhancement of the rates of subsequent cold crystallization of PLLA at high temperatures [10,11,12,13,14].

As a green processing medium, compressed CO_2_ has been utilized to increase the crystallization rate of polymers with low crystallizability such as polycarbonate (PC) [15], and PLLA [16,17,18]. The microstructure and morphology of PLLA can be tailored through controlling the crystallization and the phase transition behaviors by the aid of CO_2_ [16,19]. After the crystallization, CO_2_ can be removed from the polymer matrix by depressurization. We previously found that a glassy PLLA, which was highly stereodefective and therefore possessed extremely weak crystallizability by thermal treatment, was induced to crystallize faster by suitable treatment of high-pressure CO_2_ [18]. However, after CO_2_ treatment at 0 and 35 °C under 2 MPa, the samples remained in the glassy state due to the inadequate plasticization effect [18].

It should be noted that PLA-CO_2_ system exhibits a unique property of retrograde vitrification [17,18], which shows a glassy-to-rubbery transition by decreasing temperature from 35 to 0 °C under CO_2_ at 2 MPa due to the rapid increase of gas solubility in the polymer at lower temperatures. Therefore, it is expected that the CO_2_ conditioning at a temperature as low as 0 °C can play an important role in the formation of crystal nuclei and hence the subsequent crystallization of highly stereodefective PLLA during further annealing, which is still unclear. The mechanism by which the CO_2_ conditioning acts needs to be revealed. On the basis of previous report [18], we would like to emphasize that the short-time preconditioning by low-temperature CO_2_ under 2 MPa was insufficient to induce crystallization for a highly stereodefective PLLA with a low optical purity of 88%. As such, a two-stage crystal development method could be used to understand the enhanced formation of crystal nuclei by the low-pressure/temperature CO_2_ conditioning.

In this work, the thermal crystallization behavior of PLA after CO_2_ conditioning at 0 °C and 35 °C under 2 MPa was investigated at 110 °C via differential scanning calorimetry (DSC), polarized optical microscopy (POM) as well as time-resolved Fourier transform infrared (FTIR) spectroscopy. Despite treatment under a low pressure and temperatures, the CO_2_ preconditioning was expected to leads to non-negligible free volume expansion. Positron annihilation lifetime spectroscopy (PALS) had been used as a powerful tool for probing the nanometer-size free volume pores and holes in polymer materials. Therefore, most importantly, the effect of CO_2_ conditioning on the free volume of the samples was also further examined by PALS to elucidate the underlying microscopic mechanism and to connect the CO_2_-induced free volume change to the CO_2_ preconditioning effect on the crystallization behavior. We also compared the nucleation enhancement effect of our low temperature pressure CO_2_ preconditioning with that of physical aging.

## 2. Experimental

A commercially available polylactide (PLA) (*M*_w_ = 1.3 × 10^5^, *M*_w_/*M*_n_ = 1.9, 6% d-lactide units) with optical purity of 88%, which had been previously measured to have a stereosequence structure similar to the random copolymer of l-lactide with 12% meso-lactide [18], was provided by Zhejiang Hisun Biomaterials Co. Ltd., China (Taizhou, China). The glass transition temperature (*T*_g_) and the melting temperature (*T*_m_) of the PLA sample were determined using DSC to be ca. 56 °C and 140 °C, respectively. After drying under vacuum at 60 °C overnight, PLA films of 300 μm thickness were melt molded by compression at 180 °C for 3 min under a press of 10 MPa and then quenched in iced water. The obtained transparent films were used for compressed CO_2_ conditioning. For the measurements of POM and FTIR, amorphous films of 5–20 μm thick were prepared by casting 1 wt % PLA/dichloromethane solution on cover glass, drying in vacuum, heating at 180 °C for 3 min, and followed by quenching to 0 °C.

To prepare low-pressure CO_2_-conditioned PLA samples, the melt-quenched amorphous films were treated in CO_2_ under 2 MPa at 0 and 35 °C (labeled as PLA-T0 and PLA-T35, respectively) below the normal *T*_g_ for a fixed time of 2 h in a high-pressure vessel, which was flushed with low-pressure CO_2_ for about 3 min before pressurization. After conditioning, the vessel was depressurized slowly at an approximate rate of 0.5 MPa/min. The conditioned PLA samples were kept at ambient condition for about 3–4 weeks to ensure the total desorption of CO_2_ prior to further PALS measurements.

Isothermal cold crystallization of untreated PLA (PLA-U), PLA-T0 and PLA-T35 was studied using a TA Instruments DSC Q-2000 (New Castle, DE, USA). It should be noted that the stereodefective PLA used here has a low melting point [18], leading to a relatively narrow crystallization window as compared to optically pure PLLA. As such, these samples were heated to a fixed temperature of 110 °C at a rate of 40 °C/min, and held at 110 °C until the crystallization was complete. The time-resolved FTIR were measured with a Bruker Equinox 55 FTIR spectrometer (Bruker, MA, USA) at a resolution of 2 cm^−1^ in the wavenumber range of 4000–400 cm^−1^. The sample was sandwiched between two potassium bromide plates and was heated to 110 °C at a rate of 40 °C/min in a homemade heating cell. The spectra obtained at a constant time interval of 60 s, and 16 scans were signal-averaged to reduce the noise influence. The crystallization of samples at 110 °C was also observed by POM on an Olympus BX51 microscope (Olympus Co., Tokyo, Japan) equipped with a digital camera. The free volume of untreated amorphous (PLA-U) and CO_2_-conditioned (PLA-T0 and PLA-T35) samples was measured by PALS according to aprevious study [20], evaluating three positron lifetimes. The values of the third lifetime *τ*_3_ (ns) correspond to the pick-off annihilation of ortho-Positronium, created in PLA samples by injected positrons, and the relative intensity *I*_3_ accounts for the ortho-Positronium formation probability. All the obtained spectra were analyzed using the PALSFIT developed by Olsen, Kirkegaard, and Eldrup (Roskilde, Denmark). For each sample, tests were replicated five times, and average values are reported here.

## 3. Results and Discussion

**Figure 1:**
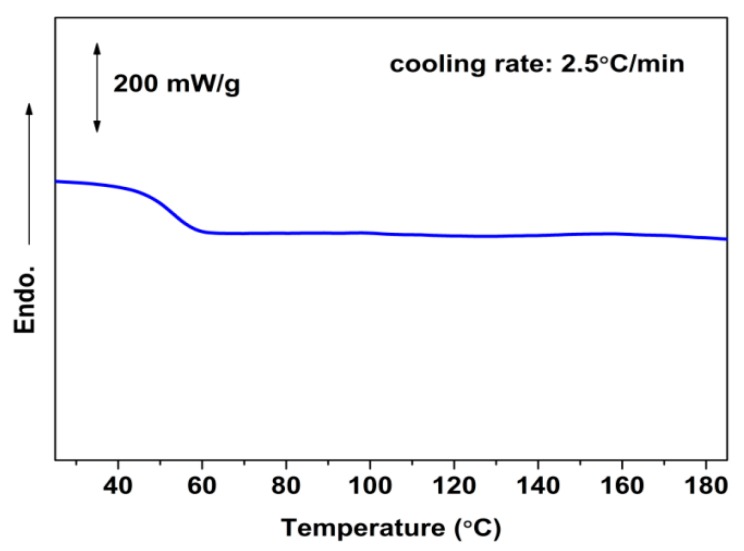
DSC cooling curves for untreated PLA sample (PLA-U) at a cooling rate of 2.5 °C/min from the melt.

**Figure 2:**
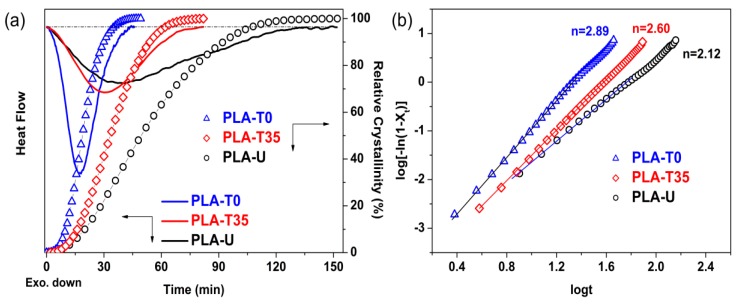
(**a**) DSC traces and variation of relative crystallinity with crystallization time for PLA-U, PLA-T35, and PLA-T0 at 110 °C and (**b**) the corresponding Avrami plots.

It is known that optical impurity greatly decreases the crystallization ability of PLLA due to the presence of stereodefective d-lactic unit in the main chains [4]. Thus, the untreated glassy PLA-U did not crystallize during DSC cooling even at a rate as low as 2.5 °C/min, as shown in Figure 1. However, the crystallization ability was unusually enhanced for the CO_2_ conditioned PLA samples (PLA-T0 and PLA-T35) even though they were just subjected to the low-pressure CO_2_ treatment at such a low temperature as 0 °C for a short time of 2 h. As shown from the DSC results for isothermal crystallization of PLA-U, PLA-T0, and PLA-T35 at 110 °C (Figure 2a), the time to complete the crystallization is reduced from about 150 min for the PLA-U to about 50 and 80 min for the PLA-T0 and PLA-T35, respectively. The crystallization kinetics was further analyzed by the well-known Avrami equation to obtain the Avrami exponent *n* and the crystallization rate constant *k* (min^−*n*^), as follows [21].

$$1-X_{t}=\;\exp\;\left(-kt^{n}\right)\;$$

where *X_t_* is the relative degree of crystallinity at time *t*, *n* is the Avrami exponent depending on the nature of nucleation and growth geometry of the crystals, and *k* is the overall crystallization rate constant. Figure 2b shows the Avrami plots of untreated and CO_2_-conditioned PLA samples crystallized at 110 °C, from which the Avrami parameters *n* and *k* can be obtained from the slopes and the intercepts, respectively, as listed in Table 1.

The value of *n* is ~2 for PLA-U, which is consistent with the crystallization behavior of neat PLLA in the literature [22]. The values of *n* are close to ~3 for CO_2_-conditioned samples in particular PLA-T0, indicating that the homogeneous crystallization mechanism and crystal growth dimensions were altered by CO_2_ preconditioning. For PLA-T0 and PLA-T35, it suggests heterogeneous nucleation and three-dimensional spherulitic growth during isothermal crystallization. On the other hand, since the unit of *k* is min^−*n*^ and *n* is not constant, it is difficult to compare the overall crystallization rate just from the *k* values. Therefore, the half-time of crystallization *t*_0.5_ = (ln2/*k*)^1/*n*^, the time required to achieve 50% of the final crystallinity of the samples, is used as a measure of gross crystallization rate. It is seen that *t*_0.5_ exhibits significant reductions of 62% and 34% from 50 min for PLA-U to 19 and 33 min for PLA-T0 and PLA-T35, respectively, also indicating the increase in gross crystallization rate. This indicates that the lower-temperature CO_2_ of 0 °C is more effective in inducing potential nulcei formation as compared with 35 °C, which agrees well the property of the retrograde vitrification of PLA/CO_2_ system [17,18], providing enhanced chain mobility at lower temperature for the formation of much more preformed nuclei.

**Figure 3:**
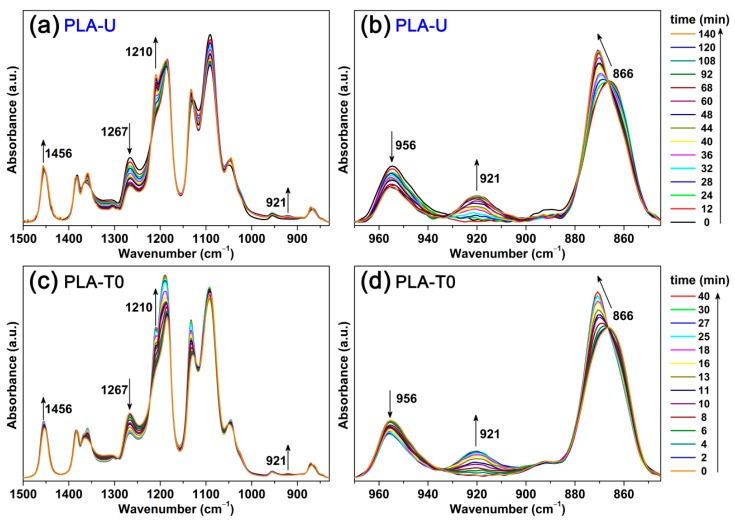
Time-resolved FTIR spectra in the 1500−830 cm^−1^ (**a**,**c**) and 970−850 cm^−1^ (**b**,**d**) ranges for PLA-U (**a**,**b**) and PLA-T0 (**c**,**d**) during the isothermal crystallization at 110 °C.

**Figure 4:**
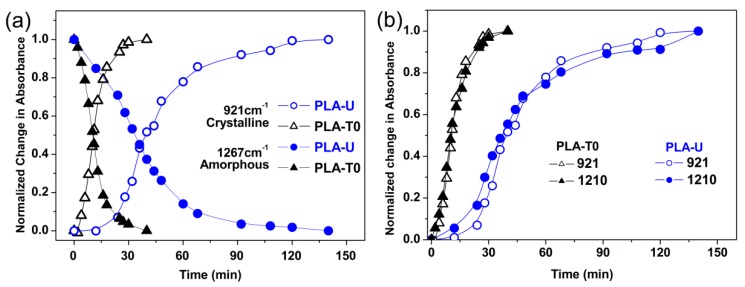
(**a**) Normalized changes in absorbance for crystalline (921 cm^−1^) and amorphous (1267 cm^−1^) bands for PLA-U and PLA-T0; (**b**) Normalized changes in absorbance for crystalline (921 and 1210 cm^−1^) bands for PLA-U and PLA-T0.

While DSC provides enthalpy measurement of crystal formation during the isothermal crystallization, FTIR offers corresponding information about conformational rearrangements of both crystalline and remaining amorphous phases. To follow the effect of CO_2_ conditioning on the conformational change during the crystallization, both the untreated (PLA-U) and conditioned PLA (PLA-T0 and PLA-T35) samples were further characterized by time-resolved FTIR. Hereafter, only the samples of PLA-U and PLA-T0 isothermally crystallized are shown for brevity. As shown in Figure 3, the crystalline-dependent bands (e.g., 1456, 1210, and 921 cm^−1^) show increased absorbance with crystallization time. Meanwhile, the 866 cm^−1^ band shifts to the higher wavenumber and becomes sharper, suggesting the formation of ordered structures [23]. On the other hand, the absorbance of the 1267 cm^−1^ band (characteristic of amorphous phase) was found to decrease monotonically with increasing time, confirming that the crystalline phase was formed at the expense of amorphous phase in both the untreated and conditioned PLA samples. Figure 4a shows the trends of normalized intensity of the bands at 921 and 1267 cm^−1^ against crystallization time, clearly indicating that the PLA-T0 shows much faster crystallization (i.e., concurrent formation/consumption in crystal/glass, respectively) kinetics, compared to PLA-U. This is in good agreement with the DSC results shown in Figure 2. Therefore, both the DSC and IR results of kinetics show that the crystallization rate has been greatly enhanced by the previous CO_2_ conditioning.

On the other hand, from the FTIR spectra, the change in interchain conformational order was further analyzed. Figure 4b shows the normalized intensities at 921 and 1210 cm^−1^ as a function of crystallization time for PLA-U and PLA-T0 during isothermal crystallization. The band at 1222 cm^−1^ corresponds to the *ν*_as_(C−O−C) + *r*_as_(CH_3_) interchain interaction, while the one at 921 cm^−1^ is sensitive to 10_3_ helix formation [24]. It can be seen that the order of the intensity changes of these two characteristic bands in PLA-T0 and PLA-U are almost similar: 1210 cm^−1^> 921 cm^−1^, indicating that the induction mechanisms of PLA crystallization in PLA-T0 and PLA-U are probably the same. That is, despite the CO_2_-induced alteration in nucleation mechanism that switched from homogeneous to heterogeneous nucleation as discussed above in DSC results, the conformational changes occurred in the induction period in PLA-U and PLA-T0 should be similar. This further implies that the low pressure CO_2_ preconditioning has played a major role in promoting the subsequent thermal crystallization at ambient pressure, which will be discussed later.

**Figure 5:**
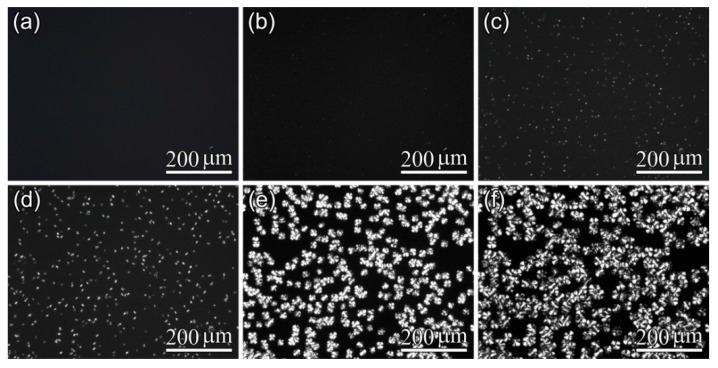
POM micrographs of PLA-U film during isothermal crystallization at 110 °C: (**a**) 0 min; (**b**) 20 min; (**c**) 30 min;(**d**) 40 min; (**e**) 90 min; and (**f**) 120 min.

**Figure 6:**
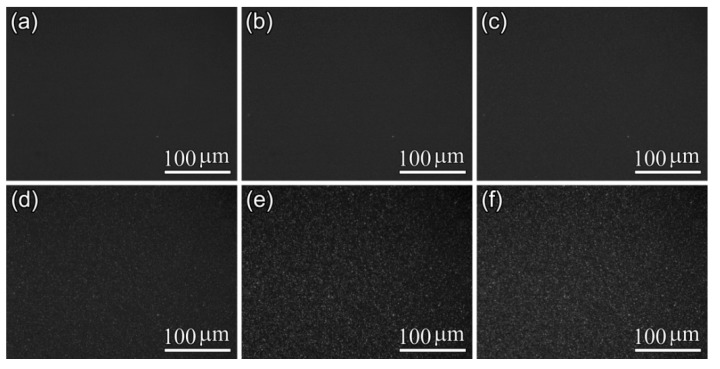
POM micrographs of PLA-T0 film during isothermal crystallization at 110 °C: (**a**) 0 min; (**b**) 6 min; (**c**) 9 min; (**d**) 13 min; (**e**) 20 min; and (**f**) 30 min.

**Figure 7:**
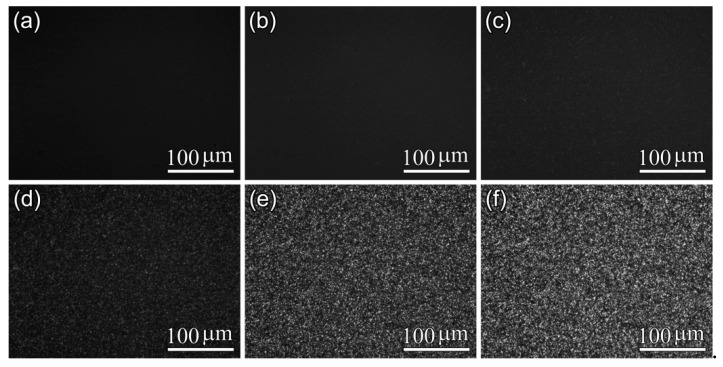
POM micrographs of PLA-T35 film during isothermal crystallization at 110 °C: (**a**) 0 min; (**b**) 6 min; (**c**) 9 min; (**d**) 13 min; (**e**) 20 min; and (**f**) 30 min.

**Figure 8:**
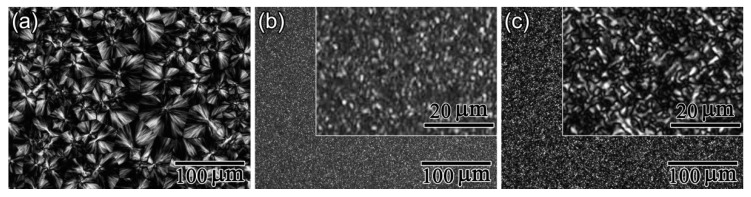
POM micrographs for fully crystallized samples of (**a**) PLA-U; (**b**) PLA-T0; and (**c**) PLA-T35. The insets in panels **b**–**c** show high-magnification images.

Furthermore, POM results shown in Figure 5, Figure 6 and Figure 7 display the influence of the previous CO_2_ conditioning on the development of the crystalline morphology of PLA-U, PLA-T0 and PLA-T35 during the crystallization. Only a very small number of tiny crystallites appeared before 20–30 min had elapsed (Figure 5c) for PLA-U, whereas a great number of birefringent grains of micron-size were observed in both PLA-T0 and PLA-T35 after a much shorter period of time of 6–9 min (Figure 6c and Figure 7c). Consequently, as seen in the POM results for full crystallized samples shown in Figure 8, large, well-developed spherulites with a size of about 100 μm appear in the PLA-U (Figure 8a), instead of the much smaller crystalline entities in PLA-T0 (Figure 8b) and PLA-T35 (Figure 8c). It clearly suggests that CO_2_ conditioning greatly promotes nucleation.

**Figure 9:**
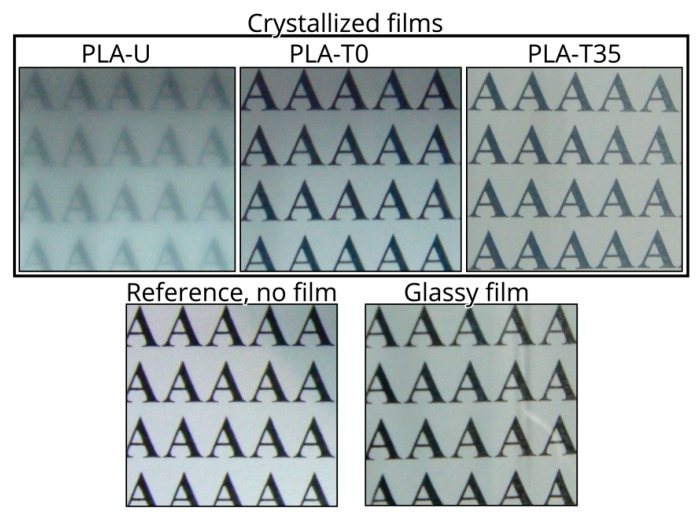
See-through (on the top on a text page) macrographs of PLA films of 20 μm thickness, fully crystallized at 110 °C. For comparison, the pictures of reference with no film and glassy one are also shown in lower row.

After crystallization, although both PLA-T0 (see inset in Figure 8b) and PLA-T35 (see inset in Figure 8c) are full of smaller birefringent crystal entities, the average size of the crystals in PLA-T0 is much finer compared to PLA-U. That is, PLA-T35 shows interwoven spherical morphology with a size of about 5–10 μm, whereas PLA-T0 contains a larger amount of grains with a much smaller size of about 1–2 μm. This definitely indicates the presence of much more preformed nuclei in PLA-T0, agreeing well the fastest kinetics of DSC and FTIR results as mentioned above. As such, as shown in Figure 9, PLA-T0 film shows high optical transparency, resembling the untreated glassy one.

In addition, the linear spherulitic growth rate of PLA-U at 110 °C calculated from Figure 5 was equal to 0.123 μm/min, which is significantly smaller than those (ca. 5 μm/min) of PLA with high optical purity (99% and above) [5]. It is noted that the growth rates of PLA-T0 and PLA-T35 are not available due to the very high nucleation density. It is well-known that the overall crystallization rate of polymers is controlled by both the density of growing nuclei/crystals and the crystal growth rate, and it has been reported that the spherulite growth rate is independent of the number of crystal nuclei [12]. Thus, it is concluded that the greatly enhanced crystallization kinetics of PLA-T0 and PLA-T35 compared to PLA-U is mostly attributed to the highly increased nucleation efficiency by the CO_2_ preconditioning, which induced the preformed nuclei. Therefore, although the crystallization ability and crystal growth rate of PLA are intrinsically weak and extremely slow, respectively, the crystallization process can finish in a short time with the help of CO_2_ preconditioning.

**Figure 10:**
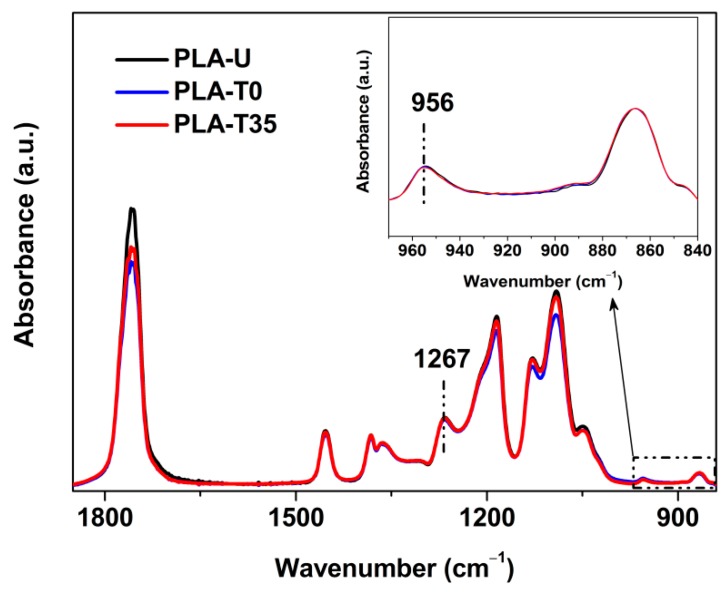
FTIR spectra in the 1850–840 cm^−1^ and 970−840 cm^−1^ (see the inset) ranges for PLA-U, PLA-T35, and PLA-T0 before further annealing.

Despite the enhanced crystallization kinetics discussed above, both PLA-T0 and PLA-T35 displayed FTIR spectra identical to that of PLA-U in its glassy state, as shown in Figure 10, definitively indicating that they were still in a glassy state after CO_2_ preconditioning. Note that, for optically pure PLLA, our previous report indicated that the mesophase with a characteristic FTIR band at 918 cm^−1^ was induced by low-temperature pressure CO_2_ under similar conditions used in this study [25]. However, the absence of the 918 cm^−1^ band for PLA-T0 and PLA-T35 in Figure 9 confirms that no mesophase in these highly stereodefective PLA samples was induced by CO_2_ preconditioning.

**Figure 11:**
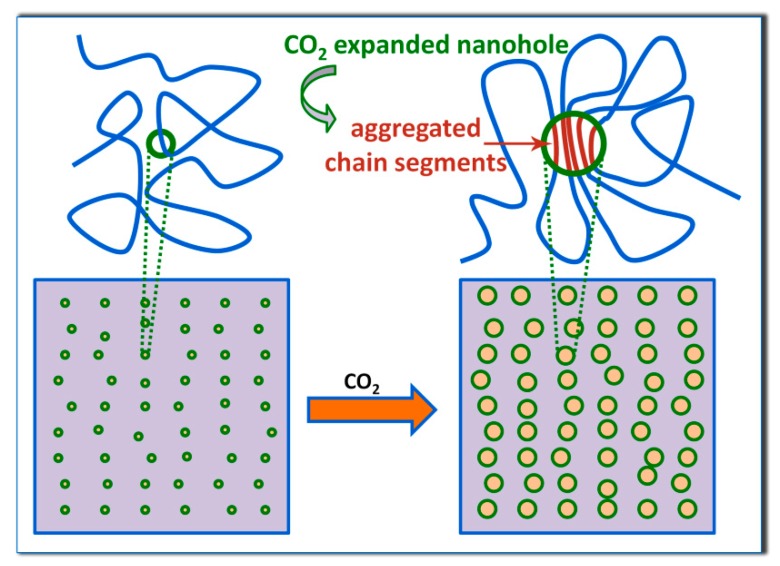
Schematic (not to scale) representation of the CO_2_ expaned nanoholes of free volume in glassy PLA samples. Upon adsorbing CO_2_, the free volume expansion was induced, leading to small clusters of parallel-aligned chain segments, which transformed to critical nuclei for inducing later crystal growth upon thermal annealing. The two boxes and holes inside them (lower row) correspond to limited parts of the samples and some free volume sites, respectively. Note that the real free volume holes are everywhere and are roughly speaking the intersegmental spaces for segmental motion, and actually a distribution of sizes exist.

It has been suggested that the formation of a local ordered structure, which accelerates the cold crystallization of PLA with relatively high optical purity, is favored by long-time physical aging around the glass transition temperature (*T*_g_) [10,11,12,13]. The PLA/CO_2_ system exhibits the property of retrograde vitrification, which has a low *T*_g_ of around 0 °C under CO_2_ at 2 MPa [17,18]. Therefore, when the PLA samples were conditioned in CO_2_ at such low pressure and temperatures, even after a short time, it is expected to induce the formation of a large amount of local ordered structures, reminiscent of those obtained by long-time physical aging. In order to provide additional insight into the mechanism, we performed the PALS measurements; the results are shown in Table 2. An increase in *τ*_3_ is observed for PLA-T0 or PLA-T35, while *I*_3_ remains substantially unchanged. This is attributed to the increase in the size of free volume holes. The radius of free volume hole (*R*) of PLA-T0 and PLA-T35 are ca. 0.287 and 0.281 nm, respectively, comparatively larger than ca. 0.277 nm of PLA-U, due to the expansion of the hole by dissolved CO_2_ during conditioning. Such an extent of the free volume expansion is non-negligible [26]. From the viewpoint of free volume theory, it is expected that a free volume expansion leads to enhanced molecular mobility. Thus, as proposed in Figure 11, owing to the adsorbed low-temperature pressure CO_2_ and thus free volume expansion, the molecular chains gained higher mobility for greater ease of re-arrangement, leading to the enhanced propensity to form small clusters of parallel-aligned molecules segments, which cannot be detected by FTIR. Note that the enhanced mobility was insufficient to induce any crystallization during CO_2_ preconditioning. Upon thermal annealing, these locally ordered domains supplied massive nucleation sites and transformed to critical nuclei for inducing subsequent PLA crystallization, provided that sufficient thermal energy was given, thus ultimately resulting in the increased overall crystallization rate.

In addition, the obtained results of *R* also account for the conditioning history/temperature dependence of the nucleation enhancement behavior and thus the thermal crystallization rate at 110 °C (Figure 2 and Figure 3). Thanks to the retrograde vitrification effect, with decreasing temperature from 35 °C to 0 °C, the decrease in the thermal energy was overcome by an enhanced plasticizing effect induced by the increase in CO_2_ solubility. Thus, a larger free volume expansion was induced in the PLA-T0, as compared to the PLA-T35, resulting in much higher mobility for forming more preformed small clusters.

We compare the possible origin of nucleation enhancement effect by our CO_2_ preconditioning with that of physical aging. Although the origin of physical aging is a long-standing subject of scientific investigations, it is reasonable to hypothesize that the physical aging is a process of densification of the glassy matrix and reduction in free volume [27,28,29]. As such, the local ordered structure is induced by densification during physical aging. In sharp contrast, after CO_2_ preconditioning in low-pressure CO_2_, the free volume was expanded while the number of nanocavities remained unchanged as evidenced by the increased *R* with almost constant *I*_3_ as shown in Table 2, leading to enhanced mobility. Moreover, limited amount of local ordered structures in optically pure PLLA were usually formed by physical aging overtime periods as long as 10^1^–10^3^ h as mentioned in the introduction. This agrees well with the observation that physical aging of polymer glass is related to the subsegmental relaxation such as the reorientation of side groups [30], resulting in a very slow relaxation occurrence toward thermodynamic equilibrium. On the contrary, in this work, despite the highly stereodefective molecular structure of PLLA, the local ordered structure can be easily induced in a much shorter period of time since the CO_2_ preconditioning afforded an atmosphere for a much higher level of relaxation associated with cooperative motion.

Finally, we emphasize that our experiments do not seek to merely enhance crystallization of PLLA by using CO_2_ preconditioning, since a cheap filler (e.g., talc) may promote its crystallization to some extent [5]. Instead, from the viewpoint of free volume, our results seek to provide new insight for understanding the CO_2_-induced ordering in PLLA. Additionally, simple low-temperature and pressure CO_2_ preconditioning greatly enhanced the formation of preformed locally order structure, which promoted the crystallization of PLA having significantly low optical purity at elevated temperatures, maintaining a neat matrix without incorporating any filler. Similar behavior was also observed for syndiotactic polystyrene, in which the CO_2_ preconditioning promoted thermal crystallization and affected the polymorphic behavior [31]. Our previous work indicated that low-pressure CO_2_-induced mesophase also greatly enhanced crystallization of optically pure PLLA [32]. However, differing from the optically pure PLLA, no trace of mesophase was found in stereodefective PLLA in this work. As such, the effect of optical purity on the formation of mesophase in PLLA needs further investigation.

## 4. Conclusions

We investigated the nucleation enhancement in a stereodefective PLLA with an optical purity of 88% by low-temperature pressure CO_2_ preconditioning. By treating at low temperatures of 0 and 35 °C, the preconditioning in low-pressure CO_2_ of 2 MPa led to greatly enhanced crystal nucleation effect in glassy PLA, thereby resulting in the accelerated crystallization rate at elevated temperatures in ambient atmosphere. The PALS results indicated that the enlarged free volume, resulted from the CO_2_ conditioning, largely accounted for the formation of locally ordered structures, providing many more nucleation sites for forming critical nuclei and hence resulting in the enhanced crystallization kinetics in glassy PLA. Particularly, when compared to the case of 35 °C, the lower temperature of 0 °C was more effective in inducing free volume expansion and thus the more locally ordered structure for significantly enhanced nucleation and crystallization too, owing to retrograde vitrification. Scientifically, we believe that this study helps in understanding the origin of the nucleation enhancement effect caused by CO_2_ preconditioning in PLA. In practice, the low-pressure CO_2_ conditioning could be used for enhancing the nucleation and thereby tailoring the semicrystalline morphology and properties of stereodefective PLA.

## Figures and Tables

**Table 1:** Summary of isothermal crystallization kinetics at 110 °C for PLA-U, PLA-T35, and PLA-T0 samples.

Sample	Summary of Crystallization Kinetics		
	*n*	*k* (min^−*n*^)	*t*_0.5_ (min)
PLA-U	2.12	1.73 × 10^−4^	50
PLA-T35	2.60	7.66× 10^−4^	33
PLA-T0	2.89	1.39 × 10^−4^	19

**Table 2:** Summary of free volume properties of PLA-U, PLA-T35, and PLA-T0 samples.

Sample	Free Volume Properties		
	τ_3_ (ns)	*I*_3_ (%)	*R* (nm)
PLA-U	1.91	15.8	0.277
PLA-T35	1.95	16.1	0.281
PLA-T0	2.01	16.0	0.287
